# High-Performance Lightweight Alloy Materials and Their Advanced Forming Technologies

**DOI:** 10.3390/ma19132859

**Published:** 2026-07-04

**Authors:** Xin Tong

**Affiliations:** 1 National Engineering Research Center of Light Alloy Net Forming and State Key Laboratory of Metal Matrix Composites, School of Materials Science and Engineering, Shanghai Jiaotong University, Shanghai 200240, China; xintong@sjtu.edu.cn; 2 Shanghai Jiaotong University Chongqing Research Institute, Chongqing 401135, China

## 1. Introduction and Special Issue Scope

Global efforts to reduce energy consumption and carbon emissions have placed lightweighting at the forefront of materials research. The transportation, aerospace, and renewable energy sectors are increasingly adopting lightweight alloys, including magnesium (Mg), aluminum (Al), and titanium (Ti) alloys, as well as low-density steels and metal matrix composites [1,2]. These materials replace conventional high-density counterparts to improve fuel efficiency, extend vehicle range, and increase payload capacity. The lightweight alloys market has experienced robust growth in recent years, reflecting the accelerating demand for weight reduction across industries.

However, each alloy system presents distinct challenges that must be overcome to realize its full potential. Mg alloys offer exceptional lightness but suffer from low absolute strength and poor heat resistance [3]. Meanwhile, their high chemical reactivity makes forming defects, particularly oxidation inclusions and casting defects, difficult to control [4,5]. These limitations have motivated the development of Mg matrix composites and advanced repair welding techniques. Al alloys research focuses on strength–ductility synergy in high-strength 7xxx and Al–Li series alloys, alongside impurity control in recycled alloys [6,7]. Ti alloys offer high specific strength but are constrained by high manufacturing costs, poor machinability, and limited tribological performance, prompting the development of Ti matrix composites [8,9]. The microstructural regulation mechanisms of low-density steels and emerging lightweight high-entropy alloys are incompletely understood and they have an insufficiently developed service performance, requiring further systematic investigation [10,11]. Addressing these issues demands the synergistic integration of alloy design, microstructural engineering, and advanced forming technologies.

This Special Issue, “High-Performance Lightweight Alloy Materials and Their Advanced Forming Technologies,” was established to provide a dedicated forum for research bridging these interconnected domains. The Issue brings together contributions on Mg, Al, Ti, and low-density steel systems, as well as their composites and bimetallic combinations, covering topics from composition optimization and precipitate control to advanced forming technologies and mechanistic understanding of deformation and failure. By highlighting recent advances and identifying persistent challenges, this Special Issue aims to accelerate the translation of lightweight alloy innovations into engineering applications, offering both fundamental insights and practical guidance for researchers and engineers working toward more sustainable, high-performance structural solutions.

## 2. An Overview of the Special Issue

Building upon the challenges outlined above, this section provides a systematic overview of the contributions in this Special Issue. Rather than presenting isolated studies, the papers are organized to show how multiple strategies, including composition optimization, process innovation, and composite reinforcement, are being deployed to tackle the core bottlenecks in each alloy system.

**Magnesium Alloys:** The three papers on Mg address the central dilemma of Mg alloys: how to overcome low strength, poor heat resistance, and processing defects while preserving their lightweight advantage. Wang et al. address the insufficient strength of as-cast Mg–Al–Zn alloys for dissolvable bridge plug applications. Through hot extrusion with a ratio of 12, they achieved simultaneous grain refinement, nanoprecipitate formation, and twin activation. Their work not only demonstrates a practical processing route for high-strength dissolvable Mg components but also clarifies the quantitative contributions of precipitation, grain boundaries, and twinning strengthening, providing a mechanistic framework for future alloy design. Gong et al. confront the weldability challenge of cast Mg–4Y–3RE–0.5Zr alloys, a critical issue given that repair welding is often unavoidable in the fabrication of large aerospace components. By systematically tailoring Y content in filler wires, they discovered that reducing Y to 2 wt.% suppresses yttria inclusions and avoids grain coarsening, achieving a joint efficiency exceeding 90%. This work establishes that non-homologous filler wire design can be a more effective strategy for defect-free Mg–RE welding. Ying et al. take a broader view, reviewing ceramic particle-reinforced Mg matrix composites as a strategy to overcome the inherent property limitations of Mg alloys. Their critical analysis of reinforcement types, fabrication routes, and strengthening mechanisms provides a roadmap for developing Mg composites with improved strength, stiffness, and high-temperature performance, while identifying interfacial compatibility as the key bottleneck requiring further attention. Collectively, these papers illustrate that Mg alloy performance can be elevated through processing control, weld metallurgy optimization, and composite strengthening.

**Aluminum Alloys:** The four Al papers converge on three major challenges: achieving strength–ductility synergy in high-strength 7xxx and Al–Li series alloys, enabling reliable welding of Al–Li alloys for aerospace structures, and controlling impurities in recycled aluminum. Xu et al. provide a comprehensive review of ultra-high-strength Al alloys for aerospace, with particular emphasis on the 7xxx series and Al–Li alloys. They systematically examine precipitation regulation, solute clustering, and thermomechanical strategies for breaking the strength–ductility trade-off and emphasize that future progress lies in heterogeneous microstructure design and machine learning-assisted alloy development. Li et al. address the weldability of advanced Al–Li alloys, increasingly used in aerospace for their low density and high specific stiffness. Their review systematically examines the formation mechanisms of equiaxed grain zones and coarse columnar grains in Al–Li welds, two microstructural features that critically degrade joint performance, and compares various control strategies including filler design, pulsed currents, and external field assistance. This work provides essential guidance for achieving reliable Al–Li alloy joints, a prerequisite for their wider adoption in lightweight aerospace structures. Le et al. tackle the Fe-rich phase problem in recycled Al–Si-Mg alloys, a pressing issue as the industry moves toward circular economy models. By introducing Cr additions, they transformed harmful needle-like β-Al_5_FeSi into blocky α-Al_15_(Fe,Cr)_3_Si_2_ with significantly reduced size and improved sphericity, achieving a 107% increase in elongation and 19% improvement in tensile strength. This work demonstrates that microalloying is an effective and practical strategy for upgrading recycled aluminum alloys. Wang et al. complement these experimental efforts by reviewing molecular dynamics simulations of bimetallic interfacial behavior, covering systems such as Al/Cu, Al/Mg, Al/Ni, Al/Ti, and Al/Fe. Their review provides an atomic-scale understanding of diffusion, intermetallic formation, and mechanical behavior at bimetallic interfaces, and identifies future directions in machine learning potentials and multi-scale simulation. Together, these papers show that Al alloy development is advancing through alloy design, welding innovation, recycling optimization, and computational characterization.

**Titanium Alloys:** Ti alloys offer outstanding specific strength but are constrained by high manufacturing costs, poor machinability, and limited tribological performance. The two papers on Ti both turn to composite reinforcement as a solution, yet they explore different processing routes that reveal complementary strengthening mechanisms. Sun et al. fabricate TiC/TC4 composites via hot isostatic pressing, achieving an excellent strength–ductility combination through uniform TiC particle dispersion and load-transfer strengthening. Their work also reveals temperature-dependent fracture mechanisms: microcracks initiate around TiC particles at room temperature due to dislocation pile-up at particle/matrix interfaces, while at elevated temperatures, crack origins shift to the matrix, accompanied by precipitation of the secondary α phase. These insights inform the design of Ti composites for high-temperature applications. Cao et al. employ laser powder bed fusion to produce TiCp/TA15 composites and demonstrate that post-heat treatment at 750 °C significantly enhances the wear resistance through promoted TiC precipitation and α + β refinement. Their work uncovers a transition in the wear mechanism from abrasive-dominant to oxidation-dominant after heat treatment, offering guidance for optimizing the tribological performance of additively manufactured Ti composites. The two papers together reveal that titanium matrix composites offer a viable pathway to overcome the limitations of conventional Ti alloys, and that processing route selection critically determines the resulting microstructure, mechanical properties, and wear resistance.

**Low-Density Steels and High-Entropy Alloys:** The remaining two papers address emerging lightweight systems that extend the scope beyond conventional Mg, Al, and Ti alloys. Mou et al. review κ-carbide precipitation in austenitic Fe–Mn–Al–C lightweight steels, a material class achieving density reduction through high Al and C additions. Their systematic review distinguishes the beneficial intragranular κ′ carbides, which enhance strength through dislocation interactions and activate deformation mechanisms, including Dynamic Slip Band Refinement, from the detrimental intergranular κ^*^ carbides that induce embrittlement. This mechanistic understanding provides a basis for composition–processing strategies to optimize the strength–ductility balance, and the review outlines future directions, including machine learning-assisted alloy design and industrial-scale thermo-mechanical processing. Huang et al. investigate the corrosion-wear behavior of (AlTiV)_100–x_Cr_x_ lightweight high-entropy alloys in 3.5 wt.% NaCl solution, addressing a critical gap in understanding how these novel materials perform under combined corrosion and wear conditions. They reveal a trade-off between hardness and corrosion resistance with increasing Cr content and identify that abrasive wear dominates material loss, while fatigue wear is exacerbated by decreased toughness and solution corrosivity. The Cr5 alloy exhibited optimal corrosion-wear resistance, outperforming TC4 by 65.5%, demonstrating the potential of AlTiVCr alloys for marine and offshore applications. Both papers address lightweight systems where precipitation control or elemental balance is key to achieving mechanical performance, highlighting the expanding frontiers of lightweight alloy research.

## 3. Conclusions

This Special Issue brings together eleven contributions that span the full spectrum of lightweight alloy research, from Mg, Al, and Ti alloys to low-density steels, high-entropy alloys, and bimetallic composites. Despite the apparent diversity of topics, a clear internal logic unites these works: each addresses a pressing challenge in lightweight alloy development and offers a processing-informed solution that advances the state of the art. The Issue covers extrusion deformation mechanisms for high-strength wrought Mg components, filler wire design for defect-free Mg–RE welding, and composite reinforcement strategies for Mg alloys. The contributions on Al alloys encompass ultra-high-strength alloy design for aerospace, the welding of Al–Li alloys, impurity control in recycled aluminum, and atomic-scale understanding of bimetallic interfaces. On Ti alloys, both hot isostatic pressing and additive manufacturing routes are explored for developing high-performance titanium matrix composites. Emerging systems including Fe–Mn–Al–C low-density steels and AlTiVCr high-entropy alloys are also addressed. Collectively, these works demonstrate that the synergy between alloy design, microstructural engineering, and advanced forming technologies is the key to overcoming the inherent limitations of each alloy system and unlocking next-generation lightweight structural solutions.

Looking ahead, the field of lightweight alloys continues to evolve rapidly, driven by emerging industrial demands and technological breakthroughs. Recent advances in areas such as ultra-large integrated die casting [12], semi-solid processing [13], additive manufacturing [14], and AI-driven alloy design [15] are opening new frontiers for lightweight applications in the automotive industry, aerospace, and beyond. These developments bring forth new fundamental scientific questions that demand urgent attention, including defect control and microstructure evolution in large-scale components, the design of cost-effective alloys compatible with emerging processing routes, the understanding of service performance under complex loading and environmental conditions, and the integration of computational modeling with experimental validation to accelerate alloy development. Addressing these challenges requires sustained interdisciplinary collaboration across materials science, manufacturing engineering, and data-driven approaches. It is with this vision that we are pleased to announce the launch of the second edition of this Special Issue, “High-Performance Lightweight Alloy Materials and Their Advanced Forming Technologies—Second Edition,” with the submission portal available at https://www.mdpi.com/journal/materials/special_issues/924T9ZE725 accessed on 28 June 2026. We invite researchers and engineers worldwide to contribute their latest findings, and we look forward to advancing this critical field together.
